# Perceived Stress, Loneliness, and Resilience in Relation to Game Addiction Among Adolescents in Bangkok During the COVID‐19 Pandemic Transition Period

**DOI:** 10.1111/jcap.70044

**Published:** 2025-11-07

**Authors:** Prangwalai Attasara, Tusana Thaweekoon, Wilai Napa

**Affiliations:** ^1^ Master of Nursing Science Program in Psychiatric and Mental Health Nursing, Ramathibodi School of Nursing, Faculty of Medicine Ramathibodi Hospital Mahidol University Thailand; ^2^ Ramathibodi School of Nursing, Faculty of Medicine Ramathibodi Hospital Mahidol University Thailand

**Keywords:** adolescents, COVID‐19 pandemic, game addiction, loneliness, perceived stress, resilience

## Abstract

**Background:**

The COVID‐19 pandemic has led to an increase in adolescent gaming due to lockdowns and the shift to online learning. Although factors linked to game addiction in adolescents before and during the pandemic have been explored, research on patterns following the easing of restrictions is limited.

**Purpose:**

This study examined the relationship between perceived stress, loneliness, resilience, and game addiction among adolescents in Bangkok during the COVID‐19 pandemic transition period.

**Methodology:**

This correlational study included 346 high school students from two schools in Bangkok, Thailand, selected using a proportional stratified random sampling method. Data were collected from November to December 2022 during Thailand's reclassification of COVID‐19 as a communicable disease under a surveillance system. Descriptive statistics and Spearman's correlation were used for the analysis.

**Results:**

The findings showed that 15.0% of adolescents were at risk of game addiction, with 4.4% being addicted. Most (87.3%) engaged in gaming, and 38.7% played games daily. Perceived stress (r_(s)_ = 0.14, *p* = 0.01) and loneliness (r_(s)_ = 0.25, *p* < 0.001) were positively correlated with game addiction, while resilience was negatively correlated (r_(s)_= −0.26, *p* < 0.001). All resilience components—“I have” (external support) (r_(s)_ = −0.21), “I am” (inner strength) (r_(s)_ = −0.28), and “I can” (interpersonal and problem‐solving skills) (r_(s)_= −0.24)—were negatively correlated with game addiction (*p* < 0.001).

**Conclusions:**

This study identified perceived stress and loneliness as risk factors for game addiction, and resilience as a protective factor. These findings offer valuable insights for professionals, including nurses, to develop targeted prevention programs for adolescents during the pandemic transition period and in the event of future crises.

## 1 Introduction

Gaming significantly influences adolescents' intellectual and emotional development. It provides entertainment and an escape from daily pressures (Melodia et al. 2020). Completing gaming missions can boost self‐esteem (Ryan et al. 2006). Moreover, gaming aids in forming adolescent relationships (Cole and Griffiths 2007) and fosters a sense of social belonging (Smahel et al. 2008). It also contributes to cognitive development and enhances creative thinking (Granic et al. 2014). Additionally, gaming allows adolescents to engage in activities that are not feasible in real life, thereby promoting autonomy (Przybylski et al. 2010). However, it is crucial to recognize that while gaming has its benefits, excessive engagement can lead to addiction and other negative consequences. Addicted adolescents experience intense focus during gameplay, frequently seek success, and require extended playtimes. They engage in challenging experiences to achieve their goals, dedicating more time and developing greater tolerance to achieve their goals. They feel discomfort when unable to play, a sign of withdrawal, and a lack of control, often playing for longer than intended. Despite recognizing the impact, attempts to reduce or stop playing the game often fail due to poor self‐control. When gaming is restricted, they exhibit irritability (American Psychiatric Association 2013).

While acknowledging the potential risks associated with excessive gaming, it is also important to recognize that gaming has evolved into a legitimate vocational and cultural pursuit in contemporary society. The rise of e‐sports, content creation on platforms such as YouTube and Twitch, and professional gaming careers represents a significant shift in how gaming should be conceptualized (Palanichamy et al. 2020). These developments suggest that high‐frequency gaming does not automatically indicate pathological behavior, as some individuals engage in extensive gaming as part of their professional development or career aspirations.

Game addiction, often referred to as Internet Gaming Disorder (IGD), significantly increased during the COVID‐19 pandemic among adolescents. Before the pandemic, the global game addiction prevalence ranged from 0.7% to 27.5%, with most estimates between 3.0% and 5.0% (Mihara and Higuchi 2017). During the pandemic, gaming time and addiction severity increased significantly. Teng et al. (2021) found that children and adolescents increased video game use, with adolescents showing a significant rise in internet gaming disorder severity compared to pre‐pandemic levels. The prevalence of problematic gaming increased from 7.4% to 11.6% during lockdowns (Cuong et al. 2021; Wang et al. 2023). Post‐pandemic studies indicate some stabilization but not a complete return to pre‐pandemic levels of activity. Han et al. (2022) noted that some adolescents returned to healthier gaming patterns after resuming school, but many continued to exhibit problematic behaviors during the lockdown period. The COVID‐19 pandemic appears to have created a lasting shift in adolescent gaming behavior, with the prevalence of problematic gaming remaining higher than pre‐pandemic levels, even after restrictions were lifted.

Game addiction affects adolescents' physical, mental, and social well‐being, leading to behavioral issues. The COVID‐19 pandemic has impacted gaming habits, as restrictions on outdoor activities have led to a more sedentary lifestyle among adolescents (Kim et al. 2023; Zheng et al. 2020). Prolonged gaming sessions contribute to physical inactivity (Haug et al. 2022) and elevate the risk of cardiovascular disease (Çelik and Bektaş 2023). Excessive gaming is associated with sleep disturbance (Fazeli et al. 2020). Additionally, gaming is linked to mental health challenges, such as anxiety, depression, and stress, which the pandemic has exacerbated (De Pasquale et al. 2021; Han et al. 2022; Kapoor and Subida 2023; Meng et al. 2024; Perez et al. 2024; Phetphum et al. 2023; Putra et al. 2023; Salerno et al. 2023; Sousa Tavares et al. 2023; Yamamoto et al. 2022). The time spent gaming may reduce the time available for studying and completing assignments, potentially diminishing students' academic performance (Anjum et al. 2024). Given the significant impact of game addiction on various aspects of adolescent well‐being, it is crucial to understand the psychological factors that contribute to or protect against its development, particularly perceived stress, loneliness, and resilience, to develop effective prevention and intervention strategies for it.

### 1.1 Perceived Stress and Game Addiction in Adolescents

Perceived stress involves evaluating events as unfavorable or having more negative than positive values, thereby threatening one's well‐being (Lazarus and Folkman 1984). When adolescents experience high levels of perceived stress, they often turn to various coping mechanisms, with online gaming emerging as a prominent one. The link between stress and gaming addiction lies in adolescents' use of games as a form of avoidance coping, where immersive virtual environments offer temporary relief from real‐world stressors such as academic pressure, interpersonal conflict, and family tension (Snodgrass et al. 2014). This distracting strategy can create a problematic cycle, as games provide immediate gratification and stress relief while allowing adolescents to avoid addressing the underlying stressors, potentially leading to addiction (Kaczmarek and Drążkowski 2014).

Before the COVID‐19 pandemic, research established a correlation between stress and game addiction, with adolescents experiencing high‐stress levels being more susceptible to addiction (Andreetta et al. 2020; Chang and Kim 2020; Yen et al. 2019). This association became more obvious during the pandemic. School closures, social isolation, and disruptions to daily routines heightened stress among adolescents while simultaneously removing traditional coping mechanisms from their lives. She et al. (2021) demonstrated that stress related to COVID‐19, particularly from schooling and online learning, was associated with depression and Internet gaming disorder, which was attributed to diminished social support, heightened academic stress, and inadequate emotional regulation. Post‐pandemic investigations suggest that while some gaming behaviors have returned to normal, the relationship between stress and gaming addiction has not completely reverted to pre‐pandemic conditions. Akdağ et al. (2023) reported no significant difference in Internet gaming disorder scores when comparing pre‐and post‐pandemic data.

### 1.2 Loneliness and Game Addiction in Adolescents

Loneliness is a subjective and distressing experience arising from a perceived gap between desired and actual social relationships, characterized by feelings of isolation and disconnection (Peplau and Perlman 1982). Adolescents often struggle to form identities and seek social connections, and this perceived deficiency can lead them to use digital games as a form of compensation. According to compensatory Internet use theory, individuals who feel lonely may turn to online gaming to alleviate negative emotions and fulfill unmet social needs (Kardefelt‐Winther 2014). Digital games provide a virtual environment where adolescents can connect, gain validation, and escape isolation, which can potentially lead to addiction when excessively used as a coping strategy.

Before the COVID‐19 pandemic, research identified an association between loneliness and digital game addiction among adolescents. Lonely adolescents often develop problematic gaming behaviors as they seek virtual interactions to compensate for deficiencies in real‐life relationships (Ekinci et al. 2019; Wang et al. 2019). Longitudinal studies have consistently confirmed that loneliness is a predictor of gaming‐related issues (Lemmens et al. 2011). This relationship is bidirectional, with gaming addiction further contributing to adolescent social isolation (Wang et al. 2019). During the COVID‐19 pandemic, social distancing measures exacerbated loneliness and increased the use of digital technology among adolescents, intensifying the relationship between loneliness and gaming addiction (Han et al. 2022; Herguner and Kaymak 2021). Post‐pandemic studies have indicated a shift in this relationship. Although gaming issues have slightly decreased, the connection between loneliness and gaming remains significant (Mohamed et al. 2023). Loneliness continues to predict game addiction, even when accounting for depressive symptoms and sleep disturbances (Hu and Xiang 2022; Mohamed et al. 2023).

### 1.3 Resilience and Game Addiction in Adolescents

Resilience, as defined by Grotberg (1995), is the human capacity to confront, overcome, be strengthened by, and transform oneself in the face of adversity. This dynamic attribute enables individuals to positively adapt to adversity and recover from challenging experiences. Research indicates that resilience serves as a protective factor against addictive behaviors, including game addiction, in adolescents. Higher levels of resilience equip adolescents with effective coping mechanisms, emotional regulation skills, and psychological strength to manage stressors without resorting to maladaptive behaviors, such as excessive gaming (Robertson et al. 2018).

Before the COVID‐19 pandemic, studies revealed a significant negative correlation between resilience and game addiction (Nam et al. 2018; Yen et al. 2019), highlighting resilience as a psychological buffer against game addiction by fostering healthier coping mechanisms in adolescents. This protective role was intensified during the pandemic. Canale et al. (2019) demonstrated that resilience moderated the relationship between perceived stress and problematic online gaming, with a more pronounced effect in those with lower resilience. The pandemic introduced stressors such as social isolation, uncertainty, and disrupted routines, which posed challenges to adolescents' mental health. Lin et al. (2021) observed that resilience continued to protect against gaming disorders, even in the face of increased pandemic stress. Post‐pandemic research suggests that the relationship between resilience and game addiction has changed. Li et al. (2024) found that resilience remains crucial but interacts more with authenticity and family closeness variables. This indicates that the protective role of resilience against game addiction has become more complex, reflecting the pandemic's enduring psychological impact.

Research has revealed concerning levels of game addiction in Thailand, with a national prevalence of 10.5% among adolescents (Hanpatchaiyakul et al. 2021) and 6.75% among those in Bangkok (Pinto 2022). The pandemic has altered digital behavior among Thai adolescents, resulting in an increase in Internet usage to approximately 10 h and 53 min per day (Seresirikachorn et al. 2022). Although extensive research has examined the relationships between perceived stress, loneliness, resilience, and game addiction among adolescents, critical gaps remain in our understanding of these relationships. First, a time‐based gap is evident in the existing literature. Prior research has documented patterns of game addiction both before the pandemic and during the lockdown. However, there is a lack of studies examining the transitional period of the pandemic. This period represents a distinctive hybrid state in which adolescents navigate between the digital habits retained during lockdowns and the resumption of in‐person activities, creating unprecedented behavioral patterns that warrant investigation.

Second, the literature highlights a significant geographical and cultural gap. Most existing research originates from Western contexts (Andreetta et al. 2020) or developed East Asian countries (Kim et al. 2022; Han et al. 2022). There is a scarcity of studies focusing on urban adolescents in Southeast Asia, who face unique stressors, including collective cultural values and the rapid pace of digital transformation, in their lives. Bangkok presents a distinctive context, characterized by Thailand's highest Internet usage rates (Electronic Transactions Development Agency 2022) and a documented 6.75% prevalence of game addiction (Pinto 2022); however, it remains underexplored during the pandemic transition period.

Third, a methodological gap remains in examining the integrated relationships between these variables during societal recovery. Although individual associations have been identified, the simultaneous interaction of perceived stress, loneliness, and resilience in influencing game addiction during the transition crisis period is unknown. This integrated analysis is essential because the pandemic may have fundamentally altered psychological dynamics. Resilience may function differently as a protective factor when traditional coping resources are restored, but digital habits persist.

Based on these identified gaps, this study aimed to evaluate the prevalence and characteristics of game addiction and explore the relationship between perceived stress, loneliness, and resilience and game addiction among adolescents in Bangkok during the COVID‐19 pandemic transition period.

## 2 Methods

### 2.1 Research Design

A descriptive correlational design was employed to examine the relationships between perceived stress, loneliness, resilience, and game addiction among adolescents residing in Bangkok. This study was conducted during the third year of the COVID‐19 pandemic (November‐December 2022), a period characterized by Thailand's transition from emergency response to endemic management. The Ministry of Public Health reclassified COVID‐19 as a communicable disease under surveillance, and schools returned to predominantly onsite learning. Data were collected from 2 secondary schools in Bangkok, operating under the Secondary Educational Service Area Office, Bangkok 1 and 2, which provide education for Grades 7–12. During the data collection process, the schools complied with the Ministry of Public Health's recommended COVID‐19 prevention measures.

### 2.2 Population and Sample

This study focused on high school students attending educational institutions in Bangkok, Thailand. The sample included 346 high school students from Grades 10–12 enrolled in the Secondary Educational Service Area Office in Bangkok during the 2022 academic year. The inclusion criteria were as follows: (1) being a male or female high school student; (2) the ability to communicate, understand, write, and read Thai; and (3) willingness to participate with parental consent. Students who withdrew from the study were excluded from the analyses.

### 2.3 Sample Size Determination

The sample size was calculated using G*Power 3.1 (Faul et al. 2009), with parameters including a Pearson's correlation coefficient, a confidence level of 0.05, a power of 0.80, and two‐tailed testing. Previous studies have indicated correlation coefficients between the variables of interest and game addiction ranging from 0.15 to 0.66. Using the most conservative estimate (0.15), the required sample size was 346. An additional 10% of the participants (*n* = 35) were recruited to account for potential incomplete responses, totaling 381. After excluding incomplete questionnaires, the final analysis included a total of 346 participants.

### 2.4 Sampling Procedure

A proportional stratified random sampling approach was employed through six sequential steps: (1) classifying schools by Secondary Educational Service Area Office (Bangkok 1 and 2); (2) randomly selecting one school from each area by drawing lots; (3) categorizing students by grade level (Grades 10–12); (4) identifying available classrooms at each grade level; (5) randomly selecting two classrooms per grade from each school, resulting in 12 total classrooms; and (6) proportionally sampling students, resulting in 179 participants from Area Office Bangkok 1 and 167 from Area Office Bangkok 2, for a total sample of 346 students.

### 2.5 Research Instruments

Data were collected using self‐administered questionnaires divided into five sections.

**The General Information Questionnaire**, developed by the researcher, was used to gather data on various demographic characteristics, including gender, age, education level, and GPA. Additionally, it collected information on living arrangements, parental marital status, relationships with parents and friends, educational experiences during the COVID‐19 pandemic, Internet usage patterns, concerns related to COVID‐19, and gaming behaviors.

**The Game Addiction Screening Test (GAST)** was developed by Pornnoppadol et al. (2014) to screen for game addiction in children and adolescents. The test consists of 16 items assessing three dimensions of gaming‐related problems: preoccupation with games (six items), loss of control (five items), and functional impairment (five items). Each item is rated on a 4‐point scale (0 = not at all, 1 = probably not, 2 = probably yes, and 3 = yes). The total score ranges from 0 to 48, with higher scores indicating more problematic gaming behaviors. The cutoff scores were ≥ 24 for boys and ≥ 16 for girls, indicating problematic game playing. The Cronbach's alpha coefficient for this test was 0.92 (Pornnoppadol et al. 2014). In the current study, Cronbach's alpha was 0.88.

**The Perceived Stress Scale (PSS)** was developed by Cohen et al. (1983) and subsequently translated into Thai by Wongpakaran and Wongpakaran (2010) to assess stress levels over the preceding month. The scale consists of 10 items rated on a 5‐point Likert scale ranging from 0 (never) to 4 (very often). Four items were positively worded and required reverse‐scoring. The total score ranges from 0 to 40, with higher scores indicating a greater perceived stress. Scores were categorized as low (0–13), moderate (14–26), or high (27–40). The scale showed a Cronbach's alpha of 0.85 (Wongpakaran and Wongpakaran 2010). In this study, Cronbach's alpha was 0.76.

**The UCLA Loneliness Scale** was developed by Russell et al. (1978) and was translated into Thai by Nahathai Wongpakaran and Tinakon Wongpakaran. This scale assesses subjective loneliness through 11 negative and 9 positive items, respectively. Each item is rated on a 4‐point Likert scale, ranging from 1 (never) to 4 (always), with reverse scoring for positive statements. The total score ranges from 20 to 80, with higher scores indicating greater loneliness. The reliability of the Thai version was indicated by a Cronbach's alpha of 0.94 (Wongpakaran and Wongpakaran 2012). The Cronbach's alpha in this study was 0.90.

**The Resilience Inventory** was developed by Nintachan et al. (2010) based on Grotberg's concept of resilience (Grotberg 1995). The test consists of 28 items to evaluate three components: “I have” (external support, nine items), “I am” (inner strength, 10 items), and “I can” (interpersonal and problem‐solving skills, 9 items). Respondents rated items on a 5‐point Likert scale, from 1 (strongly disagree) to 5 (strongly agree), with total scores ranging from 28 to 140. Higher scores indicate greater resilience. The Cronbach's alpha coefficient for this measure among Thai adolescents was 0.89 (Sangon et al. 2018). In this study, Cronbach's alpha was 0.93.

**Data Collection and Ethical Considerations**

This study was approved by the Human Research Ethics Committee, Faculty of Medicine Ramathibodi Hospital, Mahidol University (Reference Number: COA. MURA 2022/427). Written informed consent was obtained from the students and their parents/guardians. Participation was voluntary, without compensation, and with assured withdrawal rights; no academic consequences were imposed. Raw data were securely stored with restricted access using coded identifiers to ensure data confidentiality. To minimize pandemic‐related biases, data collection was conducted in accordance with COVID‐19 protocols in well‐ventilated spaces with mask requirements. Risk assessment indicated minimal psychological discomfort, and participants who experienced distress were referred to school counselors. Response bias was addressed by having participants submit anonymous questionnaires in sealed boxes. All data were destroyed after analysis, in accordance with institutional guidelines.

### 2.6 Data Analysis

Data analysis was conducted using IBM SPSS Statistics version 29, applying the following statistical methods: (1) Descriptive statistics, including percentages, ranges, means, and standard deviations, were used to assess the participants' demographic characteristics and gaming behaviors. (2) Before conducting the correlation analyses, the assumption of normality was tested using the Kolmogorov‐Smirnov test. As shown in Table 1, all study variables demonstrated significant deviations from the normal distribution (*p* < 0.05), justifying the use of nonparametric statistical methods. Therefore, Spearman's rank‐order correlation coefficient was used to examine the relationships between the variables.

**Table 1:** Kolmogorov‐Smirnov test for normality of study variables (*N* = 346).

Variables	Kolmogorov‐Smirnov Z	*p* value	Distribution
Perceived stress	0.54	< 0.05	Non‐normal
Loneliness	0.58	< 0.05	Non‐normal
Resilience	0.49	<.05	Non‐normal
Game addiction	0.95	<.05	Non‐normal

## 3 Results

### 3.1 General Characteristics of the Sample

Table 2 presents data from 346 high school students attending two schools under the Secondary Educational Service Area Office in Bangkok, Thailand. The sample comprised 40.8% males and 59.2% females, aged 15 to 18 years (Mean = 16.63, SD = 0.930). Most students (67.3%) lived with both parents, and 68.8% reported that their parents were married. Of the participants, 76.3% had good relationships with their parents, 90.4% with friends, and 54.3% had more than four close friends. Regarding academics, 56.4% reported very good grade point averages (mean = 3.40, SD = 0.522). During the first 2 years of the COVID‐19 pandemic, 67.1% of students experienced combined onsite and online learning, whereas 32.9% experienced only online learning. Most students (77.2%) participated in online education for over 6 months. By the time the data were collected, 99.1% of the students had returned to on‐site learning. Students expressed the most concern about their studies, exams, and future education (77.7%), followed by their family's economic situation (59%), and the risk of COVID‐19 infection (49.1%).

**Table 2:** Number and percentage of the sample group, classified by personal characteristics (*N* = 346).

Personal characteristics	Number	Percentage
Gender		
Male	141	40.8
Female	205	59.2
Age		
15 years old	38	11.0
16 years old	123	35.5
17 years old	114	33.0
18 years old	71	20.5
Mean = 16.63, SD =0.930, Min = 15, Max = 18		
Education levels		
Grade 10	123	35.5
Grade 11	108	31.2
Grade 12	115	33.3
GPA		
1.00–2.49 (Low to fair)	21	6.0
2.50–3.49 (Fairly good to good)	130	37.6
3.50–4.00 (Very good to excellent)	195	56.4
Mean = 3.40, SD =0.522, Min = 1.21, Max = 4.00		
Currently living with whom		
Parents	233	67.3
Relatives	46	13.3
Mother	38	11.0
Father	20	5.8
Alone	5	1.4
Friends	4	1.2
Parents' marital status		
Married	238	68.8
Divorced	54	15.6
Widowed	20	5.8
Separated	34	9.8
Current relationship with parents		
Good ‐ very good	264	76.3
Quite good	54	15.6
Quite bad ‐ bad	28	8.1
Current relationship with friends		
Good ‐ very good	313	90.4
Quite good	30	8.7
Quite bad ‐ bad	3	0.9
Numbers of close friends		
None	9	2.6
1–2 people	58	16.8
3–4 people	91	26.3
4 people and over	188	54.3
Teaching and learning management during the first 2 years of the COVID‐19 pandemic		
Online	114	32.9
Online and onsite	232	67.1
Duration of online learning		
Less than 1 month	1	0.3
1–3 months	17	4.9
3–6 months	61	17.6
More than 6 months	267	77.2
Current teaching and learning management		
Onsite	343	99.1
Online	0	0
Onsite and online	3	0.9
Purpose of internet usage (multiple answers allowed)		
Using social media such as Facebook, LINE, Instagram	340	98.3
Watching television/clips/movies/listening to music online	329	95.1
Communicating online (both calling and chatting)	314	90.8
Searching for information	295	85.3
Playing games	290	83.8
Learning online	282	81.5
Buying products online	263	76.0
Making reports	223	64.5
Reading news/articles/e‐books	204	59.0
Receiving and sending emails	191	55.2
Making financial transactions online	150	43.4
What concerns do you have regarding the COVID‐19 pandemic situation? (multiple answers allowed)		
Study, exams and further study opportunities	269	77.7
Family economy	204	59.0
COVID‐19 infection	170	49.1
Not being able to socialize	142	42.2
Not being worried	2	0.6

### 3.2 Internet and Gaming Behavior

Table 3 presents the findings regarding Internet and gaming behaviors. Participants engaged in social media (98.3%), watched videos and listened to music online (95.1%), communicated online (90.8%), searched for information (85.3%), and played games (83.8%). During the COVID‐19 pandemic, their main concerns were studies, exams, and future educational opportunities (77.7%), followed by family‐related economic issues (59%).

**Table 3:** Number and percentage of the sample group, classified by gaming behavior characteristics (*N* = 302).

Gaming information	Number	Percentage
Gaming characteristics		
Online	103	34.1
Offline	9	3.0
Online and offline	190	62.9
Number of gaming days per week		
1–2 days/week	77	25.5
3–4 days/week	68	22.5
5–6 days/week	40	13.3
Every day	117	38.7
Number of gaming hours per week		
Less than 1 h	47	15.6
1–3 h	145	48.0
4–6 h	75	24.8
7–10 h	25	8.3
More than 10 h	10	3.3
Most popular time period for playing games on weekdays (select only one answer)		
08.00 a.m. −12.00 p.m.	8	2.6
12.01 p.m. −04.00 p.m.	35	11.6
04.01 p.m. −08.00 p.m.	75	24.8
08.01 p.m. −12.00 a.m.	172	57.0
After 12.00 a.m.	12	4.0
Most popular time period for playing games on holidays (select only one answer)		
08.00 a.m. −12.00 p.m.	14	4.6
12.01 p.m. −04.00 p.m.	73	24.2
04.01 p.m. −08.00 p.m.	33	10.9
08.01 p.m. −12.00 a.m.	91	30.1
After 12.00 a.m.	22	7.3
All day	69	22.9
Gaming location		
Own home	292	96.7
Friend's home	5	1.7
At school	3	1.0
Internet café/Online gaming shop	2	0.6
Gaming devices used (can select more than one answer)		
Mobile phone	232	76.8
Computer	152	50.3
Tablet/iPad	129	42.7
Other (Nintendo Switch, Play station)	3	1.0
Persons playing games with (can select more than one answer)		
Friends	223	73.8
Alone	210	69.5
Siblings	84	27.8
Strangers	63	20.9
Boyfriend/girlfriend/partner	56	18.5
Father/mother	1	0.3
Purpose of playing games (can select more than one answer)		
Relaxation/entertainment	279	92.4
Relieving loneliness	216	71.5
Practicing problem‐solving and planning skills	110	36.4
Feeling freedom	106	35.1
Making new friends through gaming	86	28.5
Expressing oneself	72	23.8
Studying computer, animation, and graphics	57	18.9
Wanting to win against others	52	17.2
Earning money from gaming	30	9.9
Others (relieving boredom, wanting to disconnect from the outside world)	2	0.7
Types of games (More than 1 answer is possible)		
MOBA	179	59.3
MMORPG	142	47.0
First‐person shooting	129	42.7
Puzzle game	127	42.1
Simulation game	123	40.7
Adventure game	122	40.4
Action game	117	38.7
Battle royale	115	38.1
Strategy game	115	38.1
Role‐playing game	114	37.7
Sandbox	110	36.4
Survival horror	89	29.5
Music game	88	29.1
Sport game	87	28.8
Racing game	80	26.5

Among the participants, 87.3% reported having played games in the past 3 months. Most played both online and offline games (62.9%), with 38.7% gaming daily and 48% playing for 1–3 h per day. The most popular gaming times were weekdays between 8:01 p.m. and 12:00 a.m. (57% of the participants). Mobile phones were the main gaming devices (76.8%), with most participants playing at home (96.7%), either with friends (73.8%) or alone (69.5%). The main reasons for gaming were relaxation (92.4%) and alleviating loneliness (71.5%) among the participants. The most popular game genres were Multiplayer Online Battle Arena (59.3%), Massive Multiplayer Online Role‐Playing Games (47%), and First‐Person Shooting (42.7%).

### 3.3 Game Addiction

The analysis of game addiction levels (Table 4) revealed that 80.6% of participants were not addicted to games. However, 15% were classified as probably being addicted to games (problematic game playing), and 4.4% were classified as being addicted to games (problematic game playing). Gender differences were observed, with 15.6% of male participants and 22% of female participants showing some level of game addiction. The mean score for game addiction among all participants was 10.95 (SD = 8.376).

**Table 4:** Number and percentage of samples classified by average game addiction score and gender (*N* = 346).

Gender	Game addiction level		
	Not being addicted number (percentage)	Probably being addicted number (percentage)	Being addicted number (percentage)
Male	119 (84.4)	18 (12.8)	4 (2.8)
Female	160 (78)	34 (16.6)	11 (5.4)
Total	279 (80.6)	52 (15.0)	15 (4.4)

### 3.4 Perceived Stress, Loneliness, and Resilience

Most participants (70.2%) reported moderate levels of perceived stress, whereas 17.4% and 12.4% reported low and high stress levels, respectively. The mean score for perceived stress was 18.97 (SD = 5.848), indicating moderate stress. For loneliness, the mean score was 42.67 (SD = 9.953) on a scale of 20–80, suggesting moderate loneliness among participants. The mean resilience score was 108.71 (SD = 14.562) on a scale of 28–140, indicating generally high resilience. When analyzed by component, the mean scores were 35.13 (SD = 5.349) for “I have” (external support), 38.69 (SD = 5.525) for “I am” (inner strength), and 34.89 (SD = 5.111) for “I can” (interpersonal and problem‐solving skills).

### 3.5 Relationships Between Perceived Stress, Loneliness, Resilience, and Game Addiction

Spearman's rank‐order correlation analysis (Table 5) revealed that perceived stress was positively and significantly correlated with game addiction (r_(s)_ = 0.14, *p* = 0.01). Loneliness was also positively and significantly correlated with game addiction (r_(s)_ = 0.25, *p* < 0.001). In contrast, overall resilience was negatively and significantly correlated with game addiction (r_(s)_ = −0.26, *p* < 0.001). When examining resilience components separately, all three showed significant negative correlations with game addiction: “I have” (external support) (r_(s)_ = −0.21, *p* < 0.001), “I am” (inner strength) (r_(s)_ = −0.28, *p* < 0.001), and “I can” (interpersonal and problem‐solving skills) (r_(s)_ = −0.24, *p* < 0.001).

**Table 5:** Spearman's rank order correlation coefficients (r_s_) of perceived stress, loneliness and resilience with game addiction (*N* = 346).

Variables	*r*_s_	*p* value
Perceived stress	0.14	0.010
Loneliness	0.25	0.000
Overall resilience	−0.26	0.000
I have	−0.21	0.000
I am	−0.28	0.000
I can	−0.24	0.000

## 4 Discussion

### 4.1 Game Addiction in Adolescents During the COVID‐19 Pandemic Transition Period

The study's findings indicate that 19.4% of adolescents in Bangkok displayed symptoms ranging from probable game addiction (15.0%) to complete game addiction (4.4%) during the transition period. In comparison, the pre‐pandemic research by Taechoyotin et al. (2020) reported a 5.4% prevalence of internet gaming disorder among Thai secondary school students in a rural setting. This suggests a potential increase in gaming‐related issues following the pandemic, consistent with broader global trends. The unexpected finding of a higher game addiction prevalence among female adolescents (22%) than among males (15.6%) contradicts the traditional literature, which typically identifies males as having a higher risk of problematic gaming (Mihara and Higuchi 2017). However, recent research suggests that the gender gap may be narrowing, and in certain contexts, girls may show a higher risk of addiction. For example, one study found that girls had a 2.59 times greater risk of digital game addiction than boys (Gülü et al. 2023). The higher prevalence among females contradicts pre‐pandemic patterns and warrants consideration of several factors: 1) the shift toward mobile gaming during the pandemic, which traditionally attracts more gender‐balanced participation; 2) the social isolation effects potentially impacting females differently; and 3) the popularity of social‐oriented games that emphasize communication and relationship‐building, potentially appealing more to female adolescents seeking social connection during periods of restricted face‐to‐face interaction.

The findings show that 76.8% of adolescents primarily used mobile phones for gaming, which demands consideration in the context of mobile phone dependence. This gaming trend aligns with the global mobile‐first digital engagement among adolescents (Seresirikachorn et al. 2022). The widespread availability of mobile devices has created vulnerabilities to both game addiction and phone dependency (Li et al. 2022). The intersection of gaming and mobile phone use presents challenges for intervention. Unlike conventional gaming systems, which are limited by time and space, mobile phones allow for continuous access to games in various settings. This constant access to gaming may intensify addictive behaviors. Additionally, the multifunctional nature of mobile phones complicates the assessment of problematic use, as these devices serve educational and social purposes beyond just gaming.

Our finding that 38.7% of adolescents played games daily should be interpreted cautiously. Although daily gaming was associated with a higher addiction risk in some participants, it is crucial to distinguish between problematic and high‐engagement gaming, which may be purposeful or vocational in nature. The emergence of gaming as a potential career path through e‐sports, streaming, and content creation means that frequent gaming may represent skill development rather than addiction for some adolescents (Jenny et al. 2017). Future research should incorporate measures that differentiate between pathological gaming patterns and high‐frequency gaming associated with vocational aspirations or legitimate recreational engagement.

### 4.2 Perceived Stress and Game Addiction During the COVID‐19 Pandemic Transition Period

The study found a positive correlation between perceived stress and game addiction among adolescents during the COVID‐19 pandemic transition period (r_(s)_ = 0.14, *p* = 0.01). This correlation is consistent with those of previous studies. However, it presents new insights when compared with findings from pre‐ and during‐pandemic studies. Before the pandemic, research established links between stress and gaming behaviors, with gaming often serving as a coping mechanism (Király et al. 2020). Our findings suggest that this relationship continues in the post‐pandemic period, indicating that stress is a significant factor in problematic gaming behavior. These post‐pandemic findings show that this relationship persists, although potentially through different mechanisms of action. While pandemic‐era gaming was often driven by avoidance from confinement and isolation (Fernandes et al. 2020), our transition‐pandemic respondents reported engaging in gaming primarily for relaxation (92.4%) and to alleviate loneliness (71.5%), suggesting a shift from crisis‐driven escape to more normalized coping behaviors. The ongoing relationship between stress and game addiction after the pandemic may reflect the lasting changes in adolescent coping patterns that were established during the pandemic. As Hidaayah et al. (2021) noted, adolescents who develop gaming as a stress‐relief mechanism can form habitual responses that persist beyond the original stressors. The academic concerns reported by 77.7% of our sample suggest that educational pressures, which were significantly disrupted during the pandemic, continued to be a primary source of stress even after students returned to regular schools.

### 4.3 Loneliness and Game Addiction During the COVID‐19 Pandemic Transition Period

The study identified a significant positive correlation between loneliness and game addiction (r_(s)_ = 0.25, *p* < 0.001), suggesting that adolescents experiencing higher levels of loneliness are more prone to being addicted to games. This finding is consistent with both pre‐pandemic studies (Ekinci et al. 2019; Lee et al. 2019) and research conducted during the pandemic (Fernandes et al. 2020; Gao et al. 2024; Zhu et al. 2021). The results indicate that this association persists during the transition period of the pandemic. Before the pandemic, Kanat (2019) found no significant relationship between certain demographic groups. However, the current findings are consistent with those of Wang et al. (2019), who reported a positive correlation between mobile game addiction and social loneliness. In this study, 71.5% of participants reported gaming as a means to “relieve loneliness,” while 92.4% mentioned “relaxation” as their primary motivation. This suggests that while entertainment remains the primary reason, alleviating loneliness is also a significant factor.

Multiplayer online games, including Multiplayer Online Battle Arena (MOBA) and Massively Multiplayer Online Role‐Playing Games (MMORPG), are particularly addictive due to their real‐time interaction capabilities, which foster a sense of community often absent in real life (Huang et al. 2024). In this study, the preferred game types among adolescents were MOBA (59.3%) and MMORPG (47%). Several factors influenced the participants' game preferences. Cross‐platform communication extends beyond game‐specific features, with participants utilizing Discord, LINE, and Facebook Messenger to maintain social connections. Marketing and accessibility play significant roles. The free‐to‐play model in MOBA and MMORPG games, coupled with targeted marketing campaigns aimed at Southeast Asian youth, has positively impacted adoption rates. Game optimization for mobile platforms (76.8% of participants used mobile phones) has further enhanced game accessibility. The competitive gaming culture in Thailand, characterized by professional leagues for games, has elevated the status of MOBA games (Komutanont et al. 2020). Other game types, such as First‐Person Shooter (FPS) games (42.7%), Battle Royale games (38.1%), and Strategy games (38.1%), also offer multiplayer experiences, indicating that functionality alone does not account for player preferences.

Excessive online gaming can lead to social isolation as players spend more time interacting with virtual friends than with real‐life friends. This reduction in real‐life social interactions can weaken existing relationships and make it more challenging to form new ones (Prochnow and Patterson 2024). These findings underscore the need to strike a balance between online and offline social interactions. The persistence of this relationship following the reopening of in‐person learning (with 99.1% of students reporting on‐site learning) suggests the lasting effects of pandemic‐induced isolation on students' game‐playing behavior. This is concerning, as 87.3% of the participants reported gaming in the past 3 months, and 16.4% showed signs of problematic gaming. This study suggests that the relationship between loneliness and game addiction continues during the pandemic transition period, as adolescents may still rely on games for social interactions. The long‐term implications for mental health, including increased loneliness and social withdrawal, are concerning.

### 4.4 Resilience and Game Addiction During the COVID‐19 Pandemic Transition Period

The current findings revealed a significant negative correlation between resilience and game addiction (r_(s)_ = −0.26, *p* < 0.001), suggesting that adolescents with higher resilience are less likely to be addicted to games. This aligns with previous research indicating that resilience serves as a protective factor against behavioral addictions, including problematic gaming (Almulla et al. 2025; Yen et al. 2019). Grotberg (1995) emphasized that resilience is not built on a single factor but requires a combination of elements from all three categories. A resilient individual draws upon external support (“I have”), activates their inner strengths (“I am”), and applies their interpersonal and problem‐solving skills (“I can”) to overcome adversity.

The “I have” component of resilience, referring to external support and resources available to adolescents, showed a significant negative correlation with game addiction (r_(s)_ = −0.21, *p* < 0.001). This component includes supportive relationships with caring adults, including parents, family members, teachers, and friends who provide love and acceptance (Grotberg 1995). During the pandemic, adolescents faced disruptions to their support systems due to school closures, social distancing, and resource limitations (Branje and Morris 2021). These disruptions may have led them to online gaming for social connection and support (Zhu et al. 2021). Our findings indicate that in the post‐pandemic period, as traditional support systems are reinstated, adolescents with more substantial external support are less inclined to engage in problematic gaming behaviors. This observation is consistent with other studies that have demonstrated that adolescents who experience supportive family environments are less likely to engage in problematic gaming, as they feel emotionally secure and have fewer motivations to escape through gaming (Tariq and Majeed 2022; Zhu et al. 2021).

This analysis showed that the “I am” component of resilience had the strongest negative correlation with game addiction among all resilience components (r_(s)_ = −0.28, *p* < 0.001). This includes personal strengths such as self‐esteem and autonomy (Grotberg 1995). This finding aligns with previous research demonstrating that individuals with low self‐esteem were more likely to have high levels of game addiction (Chang and Kim 2020; Warburton et al. 2022). During the COVID‐19 pandemic, many adolescents experienced a decline in self‐esteem (Azmi et al. 2022), which may have led them to seek validation through online gaming. Kim et al. (2022) noted that adolescents with low self‐esteem often seek acceptance through gaming achievements. Gaming can temporarily satisfy psychological needs and provide feelings of success, fostering positive emotions when players win or receive in‐game rewards (Charoenwanit and Sumneangsanor 2014). However, this temporary boost in self‐esteem may lead to increased gaming, eventually resulting in addiction (Yang et al. 2022).

The “I can” component of resilience, which refers to social and interpersonal skills, also showed a significant negative correlation with game addiction (r_(s)_ = −0.24, *p* < 0.001). This involves effective communication, problem‐solving, emotional management, relationship building, and self‐control (Grotberg 1995). This negative correlation suggests that adolescents with less social skills are more likely to develop problematic gaming behaviors. This associates with previous research indicating that adolescents with lower social intelligence (Lee et al. 2024), emotion regulation (Eker and Taş 2022), communication skills (Kanat 2019), self‐control (Fong et al. 2024; Guo et al. 2024; Pan et al. 2024), and ineffective coping (Wu et al. 2022) are more likely to develop game addiction. Adolescents who reported using deliberate self‐control strategies, such as time‐management techniques and gaming schedules, showed better adjustment in the post‐pandemic period (Guo et al. 2024).

### 4.5 Limitations and Future Research

The chronological classification of the study period requires careful interpretation of the results. Initially, we labeled this the ‘post‐pandemic’ period based on local policy changes and the return to on‐site schooling. However, the World Health Organization declared the end of the pandemic in May 2023, suggesting that our data collection occurred during the late stages of the pandemic or the transitional phases. Future research should explore whether the patterns identified here continue into the truly post‐pandemic period.

## 5 Conclusion

The significant correlations between psychosocial factors (perceived stress, loneliness, and resilience) and game addiction highlight the importance of addressing these factors in prevention and intervention studies. These relationships should not be interpreted in isolation, but rather as an interconnected system of risk and protective factors that influence adolescent health. The data suggest a potential cascade effect: pandemic‐induced stress may have initially triggered increased gaming as a coping mechanism, which subsequently led to social isolation and increased loneliness, further reinforcing the gaming behavior. Simultaneously, adolescents with lower resilience, particularly those lacking internal strength and coping skills, are less equipped to break this cycle, resulting in a progression toward problematic gaming.

The gender‐specific findings, with higher addiction rates among females than males, challenge traditional gaming disorder paradigms and may reflect the differential impact of pandemic‐related stressors or evolving gaming preferences on addiction. This unexpected pattern warrants consideration of gender‐sensitive interpretations of how stress, loneliness, and resilience interact differently across genders in the transition pandemic context.

### 5.1 Implications for Mental Health Professionals

The COVID‐19 pandemic has highlighted the impact of health crises on adolescents' mental health by intensifying behavioral addictions. Our findings provide crucial insights for mental health professionals to better prepare for future crises. First, pandemics create a “perfect storm” of risk factors for gaming addiction. Stress, social isolation, and limited resources during the lockdown contributed to an increase in gaming‐related issues. Mental health nurses need to understand that during emergencies, monitoring gaming problems becomes critical, as protective factors such as in‐person interactions, school activities, and outdoor play may not be available. Second, the shift to online learning has transformed the way adolescents interact with technology. Mental health professionals should advocate for balanced digital policies during emergencies that maintain educational engagement while incorporating screen breaks, physical activity, and non‐digital social interactions. Third, our findings suggest that resilience should be integrated into emergency preparedness strategies to enhance overall effectiveness. The negative correlation between resilience components and gaming addiction indicates that enhancing adolescents' coping skills before crises offer protection. Schools and mental health services should develop “psychological first aid” plans to address gaming risks, including resilience workshops, crisis monitoring, and recovery programs. Finally, the persistence of gaming problems after the resumption of in‐person learning suggests that health crises may induce lasting behavioral changes. This requires extended monitoring and intervention periods rather than assuming a return to precrisis norms. Mental health services should establish tracking systems to identify adolescents with problematic gaming behaviors postcrisis and provide interventions to prevent chronic gaming addictions. These considerations emphasize that crisis preparedness must extend beyond physical health to include psychological support systems that address adolescents' vulnerabilities to behavioral addictions during social disruption.

## Author Contributions

P.A. and T.T. made substantial contributions to the conception and design, or acquisition of data, or analysis and interpretation of data; P.A. and T.T. wrote drafting the article or revised it critically for important intellectual content; P.A., T.T., and W.N. were final approval of the version to be published.

## Ethics Statement

The researcher submitted the research proposal to the institutional board of ethics committee of Ramathibodi Hospital, Faculty of Medicine Mahidol University, for approval (approval COA. MURA2022/427). The researcher explained the study objectives and participants' rights to all the students and parents. Written informed consent was obtained from both the students and their parents or guardians. Participants were informed that their participation was voluntary, that they could withdraw without consequences, and that all data would remain confidential. This manuscript is not published elsewhere and does not use AI in any portion of the manuscript.

## Conflicts of Interest

The authors declare no conflicts of interest.

## Data Availability

The data supporting this study's findings are available from the corresponding author upon reasonable request.
